# Determinants of Inadequate Complementary Feeding Among Children Aged Six Months to Two Years: A Cross-Sectional Study in Children's Hospital, Lahore

**DOI:** 10.7759/cureus.99758

**Published:** 2025-12-21

**Authors:** Sadia Butt

**Affiliations:** 1 General Practice, Allama Iqbal Medical College, Lahore, PAK

**Keywords:** 24 hr meal frequency, complementary-feeding, dietary diversity, infant’s nutrition, minimum acceptable diet

## Abstract

Background

Inadequate complementary feeding, including delayed, insufficient, or inappropriate introduction of complementary foods, remains a major contributor to infant malnutrition in low- and middle-income countries. In Pakistan, key indicators such as dietary diversity and minimum acceptable diet (MAD) remain below regional standards despite widespread breastfeeding. This study aimed to identify the factors associated with inadequate complementary feeding among infants attending an urban tertiary hospital.

Methodology

A cross-sectional study was conducted at the Pediatric Medical Department of The Children’s Hospital, Lahore, over a two-month period. A structured questionnaire was administered to 62 mothers of children aged six to 24 months. Variables assessed included parental education, healthcare utilization, income, cultural practices, and feeding behaviors. Associations between these characteristics and WHO-recommended indicators of timing of complementary feeding initiation, 24-hour dietary diversity (a child meets the Minimum Dietary Diversity if, in the past 24 hours, they consumed at least five of the following eight WHO food groups: breast milk; grains, roots, and tubers; legumes and nuts; dairy products; flesh foods; eggs; vitamin A-rich fruits and vegetables; and other fruits and vegetables), 24-hour meal frequency, and MAD were evaluated using chi-square tests with a significance threshold of p < 0.05.

Results

Among 62 children included in the study, timely initiation of complementary feeding occurred in 35 (56.5%), while 29 (46.8%) achieved minimum dietary diversity, 24 (38.7%) met age-appropriate meal frequency, and 20 (32.3%) achieved MAD. Maternal education (p = 0.082) and place of delivery (p = 0.113) showed borderline associations with timely initiation. Father’s education was significantly associated with meal frequency (p = 0.031), while antenatal care visits demonstrated a borderline association (p = 0.053). For MAD, father’s education (p = 0.056) and antenatal visits (p = 0.094) showed borderline effects.

Conclusions

The study demonstrates low dietary diversity and inadequate meal frequency among infants, with paternal education and antenatal care utilization emerging as important influencing factors. Strengthening nutrition counseling during ANC and PNC visits, improving parental education, and addressing cultural barriers may enhance complementary feeding practices and help reduce early childhood malnutrition in Pakistan.

## Introduction

Complementary feeding is defined as the gradual introduction of semi-solid or solid foods into an infant’s diet to supplement breast or formula milk and ensure adequate growth and development. A child’s health and development are strongly influenced by adequate nutrition during the first 1,000 days from pregnancy to the child’s second birthday, as noted in an unpublished report (Unpublished report: Save the Children. State of the World’s Mothers 2012: Nutrition in the First 1,000 Days (2012). Because growth is most rapid during the first year of life, proper nutrition is essential. The recommended time to initiate complementary feeding is at six months, when the infant’s gastrointestinal system is sufficiently mature to tolerate foods other than milk, as described in an unpublished report (Unpublished report: Shekar M, et al. Unpublished complementary feeding report; 2006) [1]. Complementary feeding ensures adequate nutrient intake, as breast milk or formula alone becomes insufficient after six months, a point also supported by an unpublished study (Unpublished study: Maheshwari S, et al. Unpublished complementary feeding study; 2019) [2]. Common complementary foods include mashed fruits and vegetables, as well as rice and lentil and chickpea-based preparations [3,4].

However, in low socio-economic countries, undernutrition is common due to the inadequacy of complementary feeding to meet infants’ nutritional requirements [5]. This, in turn, results in protein-energy malnutrition, impaired cognition, and hindered social development [6]. Malnutrition and nutrient deficiencies during the complementary feeding period have been reported from Pakistan and other developing countries, as described in an unpublished article (Unpublished article: Chaudhry R, et al. Malnutrition and Nutrient Deficiencies During Complementary Feeding in Developing Countries; 2007) and an unpublished conference paper (Unpublished conference paper: Vyas S, et al. Nutritional Deficiencies During Complementary Feeding in Developing Countries. International Nutrition Conference; 2014) [7].

In Pakistan, breastfeeding is widely practiced, with 94% of children having been breastfed at some point. However, only 38% of infants are exclusively breastfed for the recommended first six months [8]. The situation is even more concerning with regard to complementary feeding. Among breastfed children, only 16% achieve minimum dietary diversity, defined as the consumption of at least five food groups and the required number of meals per day. The condition is poorer among non-breastfed children, with only 10% meeting these nutritional standards. Compared to the rest of South Asia, Pakistan lags behind, as 25% of children aged 6 to 23 months achieve minimum dietary diversity and 18% receive a minimum acceptable diet, figures that exceed Pakistan’s estimates, according to an unpublished field study (Unpublished field study: Dahani A, et al. Infant and Young Child Feeding Practices in South Asia; 2020). Evidence from India similarly reports persistent undernutrition linked to inadequate feeding practices, as highlighted in an unpublished conference presentation (Unpublished conference presentation: Ramji S. Undernutrition and Infant Feeding Practices in India. National Pediatric Nutrition Conference; 2009).

Inadequate complementary feeding refers to the delayed, insufficient, or inappropriate introduction of foods, resulting in malnutrition, growth faltering, and increased susceptibility to infections [9]. A study conducted by Razia (2007) reported that delayed complementary feeding was associated with maternal education, paternal occupation, family income, parity, and family size. Similar findings were described in an unpublished article (Unpublished article: Chaudhry R, et al. Complementary Feeding Practices and Their Determinants Among Mothers of Infants; 2007). Previous studies from Pakistan and India have explored determinants of infant feeding behaviors, identifying associations with maternal education, socioeconomic status, and family size. Most available studies have been conducted in rural or community-based settings, with limited evidence from hospital-based urban populations. Furthermore, insufficient data exist on the combined influence of multiple socioeconomic, demographic, and caregiving factors within a single analytical framework, limiting understanding of how these determinants interact to affect complementary feeding adequacy.

Given this gap in the existing literature, the present study aims to identify the factors associated with inadequate complementary feeding practices among mothers of infants attending the Pediatrics Medical Department of The Children’s Hospital, Lahore. By examining a wide range of maternal, household, and caregiving characteristics within an urban hospital setting, this study provides a more comprehensive understanding of the contextual and modifiable determinants that influence complementary feeding behaviors. The findings are expected to generate evidence that can guide the development of targeted educational and nutritional interventions for mothers and caregivers, ultimately contributing to improved complementary feeding practices, the prevention of early childhood malnutrition, and enhanced child health outcomes at The Children’s Hospital, Lahore. Such efforts may include structured educational sessions for mothers, along with awareness walks and talk shows aimed at promoting appropriate complementary feeding practices.

## Materials and methods

This cross-sectional study was conducted in the Pediatrics Medical Department of The Children’s Hospital, Lahore, over a period of two months following synopsis approval. The sampling technique employed was convenience sampling, through which 62 children were recruited into the study. Inclusion criteria included healthy children aged six months to two years with inadequate complementary feeding. Exclusion criteria encompassed children with chronic illnesses, congenital anomalies, low birth weight, prematurity, or food allergies. The sample size (n = 61) was calculated at a 97% confidence level, with an anticipated population proportion of 0.85 and an absolute precision of 0.9 [10]. Although the required sample size was 61, a total of 62 participants were enrolled, as all eligible individuals meeting the inclusion criteria were retained.

All data was analyzed using IBM Corp. Released 2020. IBM SPSS Statistics for Windows, Version 26. Armonk, NY: IBM Corp. The chi-square test was employed to examine associations between categorical variables and inadequate complementary feeding. A p-value of less than 0.05 was considered statistically significant.

Following approval from the Institutional Review Board of The Children’s Hospital, Lahore, University of Child Health Sciences (Letter ID: 1141/CH-UCHS, dated 30 July 2025). A structured questionnaire was developed based on the WHO Infant and Young Child Feeding (IYCF) guidelines, and it was completed by the participants after the study purpose had been explained and informed consent was obtained. The questionnaire was designed to capture key determinants of complementary feeding practices; therefore, inter-rater or intra-rater reliability measures such as ICC or OCC were not applicable. Variables assessed included maternal education, paternal education and occupation, healthcare services utilization, household income, cultural practices, knowledge about complementary feeding, number of children, breastfeeding status, and gender of the household head. All responses were systematically documented for analysis.

Potential confounding factors included maternal knowledge and nutrition counseling quality, household food security, maternal employment status, cultural and family decision-making dynamics, maternal nutritional status and health literacy, parity and maternal age, seasonal variations in food availability, and recall or reporting bias.

Operational definitions of variables

The following operational definitions were used for variables included in the analysis. 1) Timely initiation of complementary feeding: introduction of solid/semi-solid foods at six completed months of age, as per WHO IYCF guidelines. Inadequate dietary diversity: Consumption of fewer than five out of the eight WHO-recommended food groups in the preceding 24 hours. 2) Adequate meal frequency: age-appropriate number of solid/semi-solid feedings within the past 24 hours based on WHO standards (2-3 meals for 6-8 months, 3-4 meals for 9-23 months with 1-2 snacks). 3) Minimum acceptable diet (MAD): a composite indicator combining minimum dietary diversity and minimum meal frequency; for non-breastfed children, it includes milk feeding frequency. 4) Maternal education/Paternal education: highest level of formal schooling completed (none, primary, secondary, higher). 5) Antenatal care (ANC) visits: total number of ANC contacts during pregnancy, categorized as none, 1-3 visits, or ≥4 visits. 6) Postnatal check-up: whether the mother received a health check within two months postpartum (yes/no). 7) Place of delivery: classified as home delivery or delivery at a health facility. 8) Income level: categorized as low or middle income based on self-reported household earnings. 9) Birth order: order of birth of the child (first, 2-4th, or ≥5th). 10) Number of children under 5: total number of living children in the household younger than 5 years of age. 11) Cultural beliefs affecting feeding: maternal report of any cultural or traditional practices influencing complementary feeding decisions. 12) Community illiteracy rate: maternal perception of literacy levels in her surrounding community (low vs. high). 13) Breastfeeding status: whether the child is currently breastfed, was previously breastfed but stopped, or was never breastfed.

## Results

The mean age of the children was approximately 13 ± 5 months. The mean weight and height were 8.25 ± 1.57 kg and 64 ± 10.11 cm, respectively. Among the 62 children included in the study, 45 (72.6%) were male, and 17 (27.4%) were female. Timely initiation of complementary feeding at six months was reported in 35 children (56.5%), whereas 13 (21.0%) initiated feeding late, 10 (16.1%) initiated early, and 4 (6.5%) had not initiated complementary feeding at all. Only 29 children (46.8%) met the WHO criteria for minimum dietary diversity, 24 (38.7%) achieved adequate meal frequency, and 20 (32.3%) fulfilled the criteria for the minimum acceptable diet.

Association between sociocultural factors and timely initiation of complementary feeding

The chi-square analysis (Table 1) shows no significant association between parental education, socioeconomic background, birth order, healthcare service utilization, place of delivery, number of children, cultural beliefs, breastfeeding status, and sex of the household head with the timely initiation of complementary feeding (p > 0.05). However, as presented in Table 1, maternal education (p = 0.082) and place of delivery (p = 0.113) demonstrated borderline influence, with higher maternal education and institutional deliveries associated with more timely initiation of complementary feeding.

**Table 1:** Association between parental, household, and sociocultural factors and the starting age of complementary feeding (n = 62) The association between selected parental, household, and socio-cultural factors and the starting age of complementary feeding has been presented in terms of frequency and percentage.

Variables	Categories	CF Initiated at 6 Months (n= 35)	CF Initiated After 6 Months(n=13)	CF Not Started(n=4)	CF Initiated Before 6 Months(n=10)	p-value	Chi-Square (χ²)
Antenatal care visits during pregnancy	None	3(4.8%)	1(1.6%)	0(0%)	1(1.6%)	0.764	3.348
	1-3	6(9.7%)	3(4.8%)	2(3.2%)	1(1.6%)		
	More than 4	26(41.9%)	9(14.5%)	2(3.2%)	8(12.9%)		
Postnatal checkups within 2 months postpartum	Yes	23(37.1%)	7(11.3%)	3(4.8%)	6(9.7%)	0.834	0.862
	No	12(19.4)	6(9.7%)	1(1.6%)	4(6.5%)		
Income level	Low	33(53.2%)	13(21%)	4(6.5%)	9(14.5%)	0.682	1.501
	Middle	2(3.2%)	0(0%)	0(0%)	1(1.6%)		
Education level of father	None	11(17.7%)	3(4.8%)	1(1.6%)	4(6.5%)	0.475	5.553
	Primary	20(32.3%)	9(14.5%)	3(4.8%)	3(4.8%)		
	Secondary	4(6.5%)	1(1.6%)	0(0%)	3(4.8%)		
Education level of Mother	None	8(12.9%)	3(4.8%)	0(0%)	4(6.5%)	0.082	15.328
	Primary	22(35.5%)	9(14.5%)	4(6.5%)	1(1.6%)		
	Secondary	4(6.5%)	1(1.6%)	0(0%)	4(6.5%)		
	Higher	1(1.6%)	0(0%)	0(0%)	1(1.6%)		
Place of delivery	Home	9(14.5%)	0(0%)	2(3.2%)	2(3.2%)	0.113	5.965
	Health Facility	26(41.9)	13(21%)	2(3.2%)	8(12.9%)		
Community Illiteracy Rate	Low	6(9.7%)	1(1.6%)	1(1.6%)	3(4.8%)	0.556	2.082
	High	29(46.8%)	12(19.4%)	3(4.8%)	7(11.3%)		
Number of children under 5	None	12(19.4%)	7(11.3%)	3(4.8%)	4(6.5%)	0.300	19.536
	1-2	23(37.1%)	6(9.7%)	0(0%)	6(9.7%)		
	More than 2	0(0%)	0(0%)	1(1.6%)	0(0%)		
Sex of household head	Male	34(54.8%)	13(21%)	4(6.5%)	9(14.5%)	0.562	2.052
	Female	1(1.6%)	0(0%)	0(0%)	1(1.6%)		
Birth order	1st	3(4.8%)	5(8.1%)	2(3.2%)	3(4.8%)	0.19	8.713
	2-4th	26(41.9%)	6(9.7%)	2(3.2%)	6(9.7%)		
	5th or more than 5th	6(9.7%)	2(3.2%)	0(0%)	1(1.65)		
Breastfeeding Status	Currently	11(17.7%)	2(3.2%)	1(1.6%)	4(6.5%)	0.288	7.371
	Stopped	21(33.9%)	8(12.9%	1(1.6%)	5(8.1%		
	Never	3(4.8%)	3(4.8%)	2(3.2%)	1(1.6%)		
Cultural Beliefs Influencing complementary Feeding	Yes	8(12.9%)	1(1.6%)	1(1.6%)	3(4.8%)	0.575	1.99
	No	27(43.5%)	12(19.4%)	3(4.8%)	7(11.3%)		

Association between sociocultural factors and 24-hour meal frequency

As shown in Table 2, the chi-square test indicates no significant association between parental education, socioeconomic background, birth order, place of delivery, number of children, cultural beliefs, breastfeeding status, and sex of the household head with 24-hour meal frequency (p > 0.05). However, Table 2 further demonstrates that father’s education was significantly associated with meal frequency (p = 0.031), while antenatal care visits showed a borderline association (p = 0.053).

**Table 2:** Association between selected parental, socioeconomic, and demographic factors and 24-hour meal frequency (n = 62) The association between selected parental, socioeconomic, and demographic factors and 24-hour meal frequency has been presented in terms of frequency and percentage.

Variables	Categories	Age-Appropriate Meal Frequency	Below Age-Appropriate Meal Frequency	p-value	Chi-Square (χ²)
Postnatal checkups within 2 months postpartum	Yes	19(30.6%)	20(32.3%)	0.354	0.858
	No	14(22.6%)	9(14.5%)		
Antenatal care visits during pregnancy	None	4(6.5%)	1(1.6%)	0.386	1.905
	1-3	7(11.3%)	5(8.1%)		
	More than 4	22(35.5%)	23(37.1%)		
Place of delivery	Home	8(12.9%)	5(8.1%)	0.499	0.457
	Health facility	25(40.3%)	24(38.7%)		
Income level	Low	32(51.6%)	27(43.5%)	0.479	0.501
	Middle	1(1.6%)	2(3.2%		
Education level of father	None	13(21%)	6(9.7%)	0.274	2.589
	Primary	16(25.8%)	19(30.6%)		
	Secondary	4(6.5%)	4(6.5%)		
Education level of Mother	None	8(12.9%)	7(11.3%)	0.402	2.932
	Primary	21(33.9%)	15(24.2%)		
	Secondary	4(6.5%)	5(8.1%0		
	Higher	0(0%)	2(3.2%)		
Community illiteracy rate	Low	5(8.1%)	6(9.7%)	0.569	0.324
	High	28(45.2%)	23(37.1%)		
Number of children under 5	None	14(22.6%)	12(19.4%)	0.629	0.928
	1-2	18(29%)	17(27.4%)		
	More than 2	1(1.6%)	0(0%)		
Sex of household head	Male	32(51.6%)	28(45.2%)	0.926	0.009
	Female	1(1.6%)	1(1.6%)		
Birth order	1st	7(11.3%)	6(9.7%)	0.847	0.331
	2-4th	22(35.5%)	18(29%)		
	5th or more than 5th	4(6.5%)	5(8.1%0		
Breastfeeding status	Currently	12(19.4%)	6(9.7%)	0.389	1.889
	Stopped	17(27.4%)	18(29%)		
	Never	4(6.5%)	5(8.1%)		
Cultural beliefs influencing complementary feeding	Yes	9(14.5%)	4(6.5%)	0.193	1.692
	No	24(38.7%)	25(40.3%)		

Association between sociocultural factors and 24-hour dietary diversity

As presented in Table 3, the chi-square test was used to assess the association between parental education, socioeconomic background, birth order, utilization of healthcare services, place of delivery, number of children, cultural beliefs, breastfeeding status, and sex of the household head with 24-hour dietary diversity. No significant association (p > 0.05) was identified between dietary diversity and maternal, socioeconomic, or demographic factors. However, Table 3 shows that better dietary diversity tended to occur among children of educated parents and those whose mothers had more antenatal visits, facility-based deliveries, or postnatal check-ups.

**Table 3:** Association between selected parental, socioeconomic, and demographic factors and 24-hour dietary diversity (n = 62) The association between parental, socioeconomic, and demographic factors and 24-hour dietary diversity has been presented in terms of percentage and frequency.

Variables	Categories	Inadequate Dietary Diversity (<5 Food Groups)	Adequate Dietary Diversity (≥5 Food Groups)	p-value	Chi-Square (χ²)
Postnatal checkups within 2 months postpartum	Yes	16(25.8%)	23(37.1%)	0.626	0.238
	No	8(12.9%)	15(24.2%)		
Antenatal care visits during pregnancy	None	2(3.2%)	3(4.8%)	0.053	5.871
	1-3	1(1.6%)	11(17.7%)		
	More than 4	21(33.9%)	24(38.7%)		
Place of delivery	Home	4(6.5%)	9(14.5%)	0.509	0.437
	Health facility	20(32.3%)	29(46.8%)		
Income level	Low	22(35.5%)	37(59.7%)	0.308	1.039
	Middle	2(3.2%)	1(1.6%)		
Education level of father	None	4(6.5%)	15(24.2%)	0.031	6.962
	Primary	14(22.6%)	21(33.9%)		
	Secondary	6(9.7%)	2(3.2%)		
Education level of Mother	None	5(8.1%)	10(16.1%)	0.211	4.513
	Primary	13(21%)	23(37.1%)		
	Secondary	6(9.7%)	3(4.8%)		
	Higher	0	2(3.2%)		
Community illiteracy rate	Low	5(8.1%)	6(9.7%)	0.613	0.256
	High	19(30.6%)	32(51.6%)		
Number of children under 5	None	11(17.7%)	15(24.2%)	0.667	0.81
	1-2	13(21%)	22(35.5%)		
	More than 2	0	1(1.6%)		
Birth order	1st	5(8.1%)	8(12.9%)	0.933	1.305
	2-4th	16(25.8%)	24(38.7%)		
	5th or more than 5th	3(4.8%)	6(9.7%)		
Sex of household head	Male	24(38.7%)	36(58.1%)	0.253	0.138
	Female	0	2(3.2%)		
Breastfeeding status	Currently	8(12.9%)	10(16.1%)	0.821	0.395
	Stopped	13(21%)	22(35.5%)		
	Never	3(4.8%)	6(9.7%)		
Cultural beliefs influencing complementary feeding	Yes	7(11.3%)	6(9.7%)	0.208	1.588
	No	17(27.4%)	32(51.6%)		

Association between sociocultural factors and minimum acceptable diet (MAD)

As shown in Table 4, the chi-square test was used to examine the association between parental education, socioeconomic background, birth order, utilization of healthcare services, place of delivery, number of children, cultural beliefs, breastfeeding status, and sex of the household head with the minimum acceptable diet (MAD) composite. No significant association (p > 0.05) was found between MAD and any of the assessed factors. However, Table 4 indicates that antenatal visits (p = 0.094) and father’s education (p = 0.056) demonstrated borderline associations, with higher paternal education and more antenatal visits slightly improving MAD achievement.

**Table 4:** Association between parental, socioeconomic, and demographic factors and minimum acceptable diet (MAD) among children (n = 62) The association between parental, socioeconomic, and demographic factors with minimum acceptable diet (MAD) among children has been presented in terms of frequency and percentage.

Variables	Categories	Met Minimum Dietary Diversity (Yes)	Did Not Meet Minimum Dietary Diversity (No)	p-value	Chi-Square (χ²)
Postnatal checkups within 2 months postpartum	Yes	14(22.6%)	25(40.3%)	0.425	0.637
	No	6(9.7%)	17(27.4%)		
Antenatal care visits during pregnancy	none	1(1.6%)	4(6.5%)	0.094	4.721
	1-3	1(1.6%)	11(17.7%)		
	More than 4	18(29%)	27(43.5%)		
Place of delivery	Home	3(4.8%)	10(16.1%)	0.426	0.635
	Health facility	17(27.4%)	32(51.6%)		
Income level	Low	18(29%)	41(66.1%)	O.191	1.708
	Middle	2(3.2%)	1(1.6%)		
Education level of father	None	3(4.8%)	16(25.8%)	0.056	5.772
	Primary	12(19.4%)	23(37.1%0		
	Secondary	5(8.1%)	3(4.8%)		
Education level of mother	None	4(6.5%)	11(17.7%)	0.327	3.45
	Primary	11(17.7%)	25(40.3%)		
	Secondary	5(8.1%)	4(6.5%)		
	Higher	0(05)	2(3.2%)		
Number of children under 5	None	9(14.5%)	17(27.4%)	0.758	0.553
	1-2	11(17.7%)	24(38.7%)		
	More than 2	0(0%)	1(1.6%)		
Community illiteracy rate	Low	4(6.5%)	7(11.3%)	0.748	0.103
	High	16(25.8%)	35(56.5%)		
Birth order	1st	5(8.1%)	8(12.9%)	0.849	0.327
	2-4th	12(19.4%)	28(45.2%)		
	5th or more than 5th	3(4.8%)	6(9.75)		
Breastfeeding status	Currently	5(8.1%)	13(21%)	0.889	0.236
	Stopped	12(19.4%)	23(37.1%)		
	Never	3(4.8%)	6(9.7%)		
Cultural beliefs influencing complementary feeding	Yes	4(6.5%)	9(14.5%)	0.897	0.017
	No	16(25.8%)	33(53.2%)		
Sex of household head	Male	20(32.3%)	40(64.5%)	0.321	0.984
	Female	0(0%)	2(3.2%)		

## Discussion

This study examined the timing of complementary feeding in relation to maternal, household, and community-level characteristics. Most children in the sample began complementary feeding at six months, although some were introduced to food either earlier or later, and a small group had not yet started. None of the tested associations reached conventional statistical significance (p < 0.05). However, as can be seen in Table 1, maternal education (p = 0.082), place of delivery (p = 0.113), and birth order (p = 0.190) showed borderline associations, suggesting that these factors may still influence the initiation of complementary feeding.

No significant relationship was observed between the number of antenatal care (ANC) visits (p = 0.764) or postnatal check-ups (p = 0.834) and the timely initiation of complementary feeding. These findings contrast with evidence from Ethiopia, where mothers who attended four or more ANC visits were more likely to introduce foods at the recommended age [11,12]. Similar results were documented in Nepal, where both ANC and PNC contacts increased the likelihood of timely initiation [13]. The lack of significance in the present analyses may reflect limitations in the quality or depth of nutrition counseling during maternal healthcare rather than a lack of relevance of service contact itself.

Among all factors, maternal education showed the strongest signal (p = 0.082). Mothers with primary education were more likely to introduce foods at six months (22; 35.5%) than those with no schooling or higher education, whose practices were more variable. This finding aligns with a consistent body of evidence showing that maternal education is among the most reliable predictors of timely initiation of complementary feeding [14,15]. Educated mothers tend to have greater access to health information, a better understanding of nutrition recommendations, and less influence from traditional beliefs that may lead to early or delayed initiation.

Institutional delivery was also associated with higher rates of timely initiation (26; 41.9%) compared to home deliveries (9; 14.5%), as depicted in Table 1. Although this trend did not achieve statistical significance (p = 0.113), it mirrors findings from Ethiopia, where institutional delivery has repeatedly been linked with correct timing due to opportunities for immediate counselling and advice from trained providers [12]. These results suggest that health facilities remain important contact points for reinforcing appropriate feeding practices.

Birth order showed a borderline association with the timing of complementary feeding (p = 0.190). Later-born children (second to fourth, or fifth and above) were more likely to receive complementary feeding at the recommended time than first-borns. This may reflect maternal experience and confidence acquired over successive births. Studies from Northern Ethiopia observed similar patterns, although some analyses reported no effect of parity once education and healthcare utilization were accounted for [12,15].

No significant associations were found for paternal education, income, community illiteracy, number of children under five, sex of the household head, breastfeeding status, or cultural beliefs (all p > 0.3). While qualitative studies in South Asia have highlighted cultural influences on feeding, such as early introduction of water or delayed introduction of semi-solids, quantitative evidence linking these factors directly to timely initiation has been less consistent [16].

In this study, none of the maternal, household, or service-related variables demonstrated a statistically significant association with meeting the minimum dietary diversity (MDD-8) indicator, defined as consumption of at least five of the eight WHO-recommended food groups within the preceding 24 hours. All tested covariates showed p-values > 0.05. The lowest p-value was observed for cultural beliefs (p = 0.193), followed by father’s education (p = 0.274). Other factors, including postnatal check-ups (p = 0.354), antenatal care (p = 0.386), place of delivery (p = 0.499), maternal education (p = 0.402), income level (p = 0.479), community illiteracy (p = 0.569), number of children under five (p = 0.629), sex of household head (p = 0.926), birth order (p = 0.847), and breastfeeding status (p = 0.389), were clearly non-significant.

No association was found between dietary diversity and maternal health service utilization indicators, namely ANC, postnatal care (PNC), or place of delivery. For example, among mothers with four or more ANC visits, 23 (37.1%) of children achieved MDD compared to 22 (35.5%) who did not, while dietary diversity was nearly evenly split among children delivered in health facilities (24; 38.7% meeting MDD vs. 25; 40.3% not meeting). These null findings contrast with evidence from Bangladesh, where nationally representative analyses demonstrated that ≥4 ANC visits and facility-based delivery were both significantly associated with improved dietary diversity. Similarly, studies from Ethiopia reported that both ANC attendance and health facility delivery increased the likelihood of children achieving MDD [17-19].

In this study, maternal education (p = 0.402) and household income (p = 0.479) showed no significant association with dietary diversity. Father’s education, while showing the second lowest p-value (p = 0.274), also failed to reach significance. This finding is supported by another study in which maternal education did not influence complementary feeding practice [20]. However, these results diverge from the broader literature, where maternal education and household wealth are among the strongest determinants of child dietary diversity. Evidence from Pakistan, using a large sample of 18,699 children, demonstrated that dietary diversity was significantly higher among children of educated mothers and in wealthier households; urban residence and, to a lesser extent, prenatal care also favored achieving MDD [14]. Nationally representative analyses from Ethiopia similarly identified maternal education and household wealth, along with community-level influences, as key predictors of dietary diversity [15]. Analyses of the Bangladesh Demographic and Health Survey also revealed clear pro-rich inequalities in MDD, underscoring the socioeconomic gradients that shape feeding practices [18]. The absence of such associations in the current study is likely attributable to small cell sizes, especially for middle-income households and higher maternal education, which reduced statistical power to detect effects that are consistently observed elsewhere.

Community illiteracy (p = 0.569), number of children under five (p = 0.629), and sex of the household head (p = 0.926) were not associated with dietary diversity. Similarly, birth order (p = 0.847) and breastfeeding status (p = 0.389) showed no significant effect. The lowest p-value observed in this category was for cultural beliefs influencing complementary feeding (p = 0.193), suggesting a possible but non-significant influence. Although children from households reporting no cultural restrictions on complementary feeding were more likely to meet MDD than those reporting cultural barriers, the association did not achieve statistical significance. Prior research supports the importance of broader sociodemographic and economic factors over household composition in predicting dietary diversity. For instance, analyses from Nepal have shown that wealth, maternal education, and urban residence are stronger determinants of MDD than parity or household headship [21]. Regional reviews from Ethiopia and sub-Saharan Africa similarly emphasize the dominant role of maternal education and wealth over structural household variables [15,22].

Father’s education showed a significant association with minimum meal frequency (MMF) (p = 0.031). Households where fathers had secondary education displayed a more favorable distribution (age-appropriate 6/62 ≈ 9.7% vs. below 2/62 ≈ 3.2%), whereas no schooling or only primary schooling clustered more in the below-age-appropriate group (e.g., none: 15/62 ≈ 24.2% below). This pattern is consistent with evidence from South Asia showing that paternal education contributes to complementary feeding adequacy, likely via improved income, health literacy, and decision-making alongside maternal factors [20].

Antenatal care (ANC) visits displayed a borderline association with MMF (p = 0.053). Children of mothers with four or more ANC contacts had higher absolute counts in both MMF categories (age-appropriate 21; 33.9% vs. below 24; 38.7%) compared with those with fewer contacts, indicating exposure without uniformly correct practice. Multi-setting studies report that structured nutrition counseling embedded within ANC and PNC increases the odds of meeting MMF, but effects vary with counseling quality, intensity, and local food access [23,24]. The borderline result here suggests scope to strengthen counseling content and follow-up. In contrast, postnatal check-ups (within two months) were not associated with MMF (p = 0.626). Despite their proximity to the complementary feeding window, such contacts may be brief and focused on immediate postnatal issues rather than feeding demonstrations; however, previous studies have emphasized the importance of PNC in achieving MMF [23].

Similarly, the place of delivery was not associated with MMF (p = 0.509). Although several studies link institutional delivery with improved IYCF, especially MAD, the effect on MMF alone is context-dependent and tends to emerge when discharge counseling is strong and reinforced within the community [25]. Household income showed no statistical association (p = 0.308), although more children from low-income households fell below MMF (59.7%). Community illiteracy (p = 0.613) and the number of children under five (p = 0.667) were also not associated. Elsewhere, community-level literacy and parity can influence MMF through social norms and resource competition, but effects are heterogeneous and often attenuate after adjustment (Indicator guide: INDDEX Project, Tufts University). Minimum Acceptable Diet (MAD-IYCF) Indicator Description. 2022) [26]. Birth order (p = 0.933) and sex of the household head (p = 0.321) were not associated, which contrasts with existing evidence suggesting that in urban settings, second- to fourth-born children are more likely to meet MMF [27,15], and that male-headed households may be more prone to achieving MMF [23].

This is supported by another study showing that adequate complementary feeding is more likely in wealthier socioeconomic classes compared with middle-income families [20]. Mother’s education did not reach significance (p ≈ 0.211), but the distribution was directionally favorable at the secondary level (age-appropriate 6; 9.7% vs. below 3; 4.8%). Large multi-country analyses show that maternal education robustly predicts IYCF adequacy, including MMF, through knowledge, agency, and service uptake [25,10]. The non-significant findings here likely reflect sample size rather than a true absence of effect.

Breastfeeding status (p = 0.821) also did not relate to MMF in this dataset, although several analyses have shown interactions between continued breastfeeding and complementary feeding frequency by age, counseling exposure, and food security [23]. Finally, cultural beliefs were tabulated without a reported p-value, and descriptively, no clear gradient was observed. Prior literature, however, documents context-specific effects, including taboos on certain foods and perceptions of satiety, but quantifying their independent contribution requires larger samples and validated scales (Guideline: World Health Organization. Infant and Young Child Feeding: Normative Recommendations on Frequency and Variety. 2023) [27].

In this study, only 20 (32.3%) children achieved the minimum acceptable diet (MAD), and none of the covariates tested showed a statistically significant association at p < 0.05. However, two variables, father’s education (p = 0.056) and number of ANC visits (p = 0.094), emerged with borderline significance, suggesting potentially important roles in influencing complementary feeding practices. Other factors, including postnatal check-ups (p = 0.425), place of delivery (p = 0.426), income level (p ≈ 0.191), maternal education (p = 0.327), number of under-five children (p = 0.758), community illiteracy (p = 0.748), birth order (p = 0.849), breastfeeding status (p = 0.889), cultural beliefs (p = 0.897), and sex of household head (p = 0.321), showed no significant associations. MAD, as defined by the World Health Organization, incorporates both minimum dietary diversity and minimum meal frequency, with an added requirement of milk feeding for non-breastfed children (Technical Metadata: World Health Organization. Child Feeding: Minimum Acceptable Diet 6-23 Months [GHO Metadata] 2025). The association between ANC visits and MAD showed a dose-response trend, with higher ANC exposure linked to greater attainment of MAD, although the relationship did not reach statistical significance. This finding is consistent with evidence from Bangladesh, where ≥4 ANC visits significantly increased the odds of achieving MAD (AOR 1.74), and from Ghana, where ANC and postnatal contacts were strong predictors of MAD [18,19]. The non-significant results here may therefore reflect limited statistical power rather than a true absence of effect. Similarly, the association between institutional delivery and MAD was favorable but non-significant, aligning with findings from Ethiopia and other sub-Saharan African settings where facility births increased the likelihood of achieving MAD due to enhanced counseling opportunities around the time of delivery [22].

Parental education emerged as another potentially important determinant. Father’s education had the lowest p-value (p = 0.056) and showed a clear gradient, suggesting that higher paternal schooling may support better dietary practices. This echoes findings from Pakistan, where the father’s education significantly doubled the odds of meeting MAD in multivariable models [20], and from Ghana, where both maternal and paternal education were positively associated with MAD [19]. Maternal education in our sample showed a positive but non-significant trend, similar to national analyses in Pakistan, which report inconsistent associations between maternal schooling and MAD after adjustment [20]. In contrast, large-scale studies from Bangladesh and sub-Saharan Africa consistently identify maternal education as a robust predictor [18,10], highlighting the importance of sample size and contextual variation in determining its effect.

Economic status was not significantly associated with MAD in our data, although the direction was consistent with literature from multiple low- and middle-income countries. In Bangladesh and sub-Saharan Africa, wealth gradients have been strongly associated with MAD attainment [18,10]. The lack of significance here is likely due to the limited sample size and small numbers in higher-income categories. Similarly, factors such as breastfeeding status and household headship did not show significant associations, although regional and multi-country analyses often identify these as contributors to dietary adequacy [20,10].

This study offers some strengths. By using four WHO-recommended indicators, timing of complementary feeding initiation, 24-hour dietary diversity, 24-hour meal frequency, and the minimum acceptable diet (MAD), it provides a broad and balanced picture of complementary feeding practices. The reliance on standardized indicators also allows comparison of these findings with regional and international evidence. Another strength lies in the breadth of determinants explored, ranging from parental education and household characteristics to health service use and cultural influences, offering a more holistic view of the factors shaping child-feeding practices in this context. Situating the results within both regional and global literature further adds to the relevance of this work, providing much-needed evidence from Pakistan, where research on this topic remains limited.

At the same time, several limitations should be acknowledged. The cross-sectional design means the study can demonstrate associations but cannot establish causality. The relatively small sample size may have limited statistical power, particularly for variables showing borderline significance, such as maternal education, paternal education, and antenatal care (ANC) visits. Data on complementary feeding practices relied on maternal recall of the previous 24 hours, which is susceptible to both recall error and social desirability bias. Although dietary diversity was assessed using WHO’s food-group method, the measure does not account for portion sizes, nutrient density, or frequency within food groups, which may have led to an incomplete picture of dietary adequacy.

Some potentially important factors, such as household food security, seasonal variations in food access, maternal knowledge, and the quality of nutrition counseling during ANC and postnatal care (PNC), were not captured. The study was also conducted in a single community, which may limit generalizability to other regions. Moreover, because most participants came from low-socioeconomic backgrounds with limited income and education, the sample lacked diversity, restricting the ability to fully explore how socioeconomic status shapes feeding outcomes. Finally, while healthcare utilization was assessed in terms of ANC and PNC visits, the quality and content of counseling during these visits were not evaluated, which may explain why service contact alone did not show strong associations with feeding practices.

The results of this study carry several important implications for improving complementary feeding practices in Pakistan and other low- and middle-income countries (LMICs). One key finding was the influence of parental education and ANC attendance. While maternal education showed a borderline effect, paternal education was significantly associated with meal frequency. This underlines the need for programs and policies that involve both parents, rather than focusing solely on mothers, in efforts to promote optimal infant and young child feeding (IYCF) practices.

The findings also highlight gaps in the current use of health services. Although mothers had contact with ANC and PNC, this alone did not guarantee improved feeding outcomes. This suggests that the content and delivery of counseling during routine health visits need to be strengthened. Practical, culturally relevant advice provided consistently by frontline health workers could help families translate knowledge into better feeding behaviors.

Another concern is the persistently low achievement of dietary diversity and MAD. These gaps reflect broader challenges of food insecurity and poverty, which cannot be addressed by the health sector alone. Tackling them will require multifaceted action, including targeted social protection measures, food fortification initiatives, and community-based programs supporting household food security.

Even though cultural beliefs did not reach statistical significance in this study, households where such beliefs influenced child feeding were less likely to achieve adequate dietary diversity. This highlights the need for behavior change communication (BCC) strategies that actively engage communities, address myths and taboos, and encourage the inclusion of diverse, nutrient-rich foods in young children’s diets.

## Conclusions

The analysis shows that although most associations were not statistically significant, consistent patterns emerged across complementary feeding indicators. Maternal and paternal education, place of delivery, and antenatal care utilization appeared to influence feeding practices even when they did not reach statistical significance.

Overall, dietary diversity and Minimum Acceptable Diet (MAD) levels were notably low, underscoring gaps in both knowledge and implementation of recommended practices. These findings highlight the need for targeted, culturally informed interventions that engage both parents and strengthen nutrition counseling within existing maternal health services. Integrating structured nutrition counseling into routine ANC and PNC visits may provide a practical and effective approach to improving complementary feeding practices.

## Appendices

Data collection questionnaire

Factors Associated With Inadequate Complementary Feeding

This questionnaire is based on WHO Infant and Young Child Feeding (IYCF) guidelines and key determinants identified in peer-reviewed research. It is intended to help assess risk factors linked with inadequate complementary feeding among children aged 6-24 months.

Infant Identification and Anthropometric Information

Table 5 provides a structured template for recording the basic identification and anthropometric details of each infant included in the study. The parameters listed, such as name, age in months, gender, birth weight, current weight, and height, were collected to establish baseline characteristics and assess growth patterns. These details support accurate classification of nutritional status and ensure that individual-level information is systematically documented for analysis.

**Table 5:** Infant identification details

Parameter	Response
Infant Name	Enter Response: ____________________________________
Age (in months)	Enter Response: ____________________________________
Gender	Enter Response: ____________________________________
Birth Weight (kg)	Enter Response: ____________________________________
Current Weight (kg)	Enter Response: ____________________________________
Height(cm)	Enter Response: ____________________________________

Comprehensive Complementary Feeding Assessment Questionnaire 

Table 6 presents the structured questionnaire used to collect detailed information on complementary feeding practices and associated maternal, household, and environmental factors. The tool is divided into thematic sections, including infant feeding practices, maternal health service utilization, socioeconomic characteristics, household demographics, and cultural or environmental influences. Each indicator includes predefined response options and corresponding notes to ensure consistency in data collection and to guide interpretation of risk levels (e.g., feeding adequacy, healthcare utilization patterns, and educational status). This comprehensive questionnaire served as the primary instrument for assessing determinants of dietary diversity, meal frequency, Minimum Acceptable Diet (MAD), and timing of complementary feeding within the study population.

**Table 6:** Comprehensive data collection questionnaire for complementary feeding study

Section	Indicator / Question	Response Options	Notes / Comments
Infant Feeding	Age at introduction of solids/semi-solids (months)	____ months	Inadequate if <6 or >8 months
	24-h Dietary Diversity: Food groups fed yesterday	☐ <5 groups	Adequate: ≥5 out of 8 food groups.
		☐ ≥5 groups	
	24-h Meal Frequency (breastfed child)	☐ Below age-appropriate	Recommended feeding frequency: 6–8 months: 2–3 meals + 1–2 snacks 9–11 months: 3 meals + 1–2 snacks 12–24 months: 3 meals + 2–3 healthy snacks
		☐ Adequate	
	Minimum Acceptable Diet (MAD) composite	☐ No	No = Inadequate feeding
		☐ Yes	Yes = Adequate Feeding
Maternal Health & Services	Postnatal check-up within 2 months postpartum	☐ Yes	Yes: Done
		☐ No	No: No Follow up
	Antenatal care visits during pregnancy	☐ 0	High Risk
		☐ 1–3	Moderate Risk
		☐ ≥4	Less Risk
	Place of delivery	☐ Home	Home = High risk
		☐ Health facility	Health facility = Low risk
Socioeconomic Factors	Household wealth / income level	☐ Low	Low: Daily wages
		☐ Middle	Middle: Stable source
		☐ High	High: Luxurious lifestyle
	Maternal education level	☐ None	No Education
		☐ Primary	Primary: Grade 1-5
		☐ Secondary& above	Secondary: Grade 9-12 & above
	Paternal education level	☐ None	Higher risk
		☐ Primary	Low Risk
		☐ Secondary	Less Risk
Household Characteristics	Number of children under 5 years	☐ 0	None
		☐ 1–2	Less than 3
		☐ >2	More than 2
	Sex of household head	☐ Male	Head is Male
		☐ Female	Head is Female
	Community illiteracy rate	☐ Low	Low: Low Risk
		☐ High	High = Higher risk
Child Factors	Child’s age (months)	____ months	Younger infants = higher risk
	Birth order	☐ 1st	Firstborns often higher risk
		☐ 2nd–4th	Less Risk
		☐ ≥5th	Lower Risk
	Breastfeeding status	☐ Currently BF	Continued BF improves adequacy
		☐ Stopped	Stopped BF
Environmental & Cultural Factors	Cultural beliefs/practices affecting CF	☐ Yes, specify: __________	Yes: Affected
		☐ No	No: Not Affected

Cultural Beliefs and Taboos Affecting Complementary Feeding

Table 7 summarizes common cultural taboos and beliefs that influence complementary feeding practices in Pakistan. These practices, such as delaying the introduction of solids, avoiding nutrient-rich foods like meat, eggs, and fish, or relying solely on liquids or traditional remedies, can contribute to inadequate dietary diversity and delayed nutritional progression in infants. The table highlights how misconceptions about digestion, safety, seasonal restrictions, and family food practices may hinder adherence to WHO-recommended feeding guidelines. Understanding these cultural beliefs is essential for designing effective community-based nutrition counseling and behavior-change interventions.

**Table 7:** Cultural beliefs associated with complementary feeding in Pakistan

Taboo	Description
Delaying solids until the first tooth appears	Belief that babies can’t digest food until they get teeth.
Avoiding meat and eggs	Considered “heavy” or “hot” foods for infants.
Withholding fruits or vegetables	Fear of diarrhea or colic.
Only feeding liquids (like rice water or tea)	Seen as easier to digest, even when babies need solids.
Belief that breast milk alone is sufficient beyond 6 months	Delays introduction of solid foods despite WHO guidelines.
Avoiding fish or certain foods during specific months (e.g., summer)	Cultural seasonal restrictions without scientific basis.
Belief that spicy or family food causes harm	Avoidance of family meals, even in mashed form.
Introduction of honey early	Unsafe due to risk of botulism but used traditionally.
Feeding only specific foods like cerelac or khichdi repeatedly	Belief that variety may harm the child’s digestion.
Using herbal tonics or home remedies instead of real food	May delay proper feeding and nutritional adequacy.
